# Study on the Extraction of Rare Earth Elements (REEs) from Phosphogypsum Using *Gluconobacter oxydans* Culture Solution

**DOI:** 10.3390/molecules30030674

**Published:** 2025-02-03

**Authors:** Jiangang Zhang, Zhuo Qi, Zijian He, Xinyue Zhang, Qinglian Zhang, Xiangdong Su

**Affiliations:** 1 Key Laboratory of Light Metal Materials Processing Technology of Guizhou Province, Guizhou Institute of Technology, Guiyang 550025, China; zhangjg009@126.com (J.Z.); hezj@git.edu.cn (Z.H.); 2 College of Matterials and Metallurgy, Guizhou University, Guiyang 550025, China; 18585398445@163.com; 3 School of Chemical Engineering, Guizhou Minzu University, Guiyang 550025, China; 15057928600@163.com; 4 School of Biology and Environmental Engineering, Guiyang University, Guiyang 550005, China; zql_emoji@163.com

**Keywords:** *Gluconobacter oxydans*, phosphogypsum, rare earth elements, bioleaching

## Abstract

With the rapid development of modern industry, particularly in the fields of electric vehicles and renewable energy technologies, the demand for rare earth elements (REEs) has surged dramatically. Phosphogypsum (PG), which is an industrial waste product generated during the production of phosphoric acid through the sulfuric acid process, is rich in REEs. However, traditional chemical leaching methods pose environmental pollution and resource wastage issues. This study aims to explore the feasibility and optimal conditions for bioleaching REEs from phosphogypsum using *Gluconobacter oxydans* (*G. oxydans*). The phase composition and components of phosphogypsum, as well as the growth characteristics and leaching efficiency of *G. oxydans*, were analyzed in detail using SEM, EDS, XRD, and XRF techniques. Experimental results indicate that *G. oxydans* can effectively leach REEs from phosphogypsum under conditions of 28 °C, an agitation speed of 150 rpm, and a liquid-to-solid ratio of 4:1, with a maximum leaching efficiency of 24.67%. Moreover, it is revealed in the study that *G. oxydans* exhibits selectivity in leaching REEs. Specifically, the leaching efficiency for Nd is significantly enhanced at low pH values. This research provides a theoretical basis and practical application example for the efficient and environmentally friendly recovery of REEs from phosphogypsum.

## 1. Introduction

Against the backdrop of accelerating technological advancements, the contemporary industrial sector is undergoing profound transformations, notably within the domains of electric transportation and clean energy technologies. These groundbreaking developments have given rise to a marked surge in the demand for rare earth elements (REEs), which play a pivotal role in enhancing the capabilities of energy storage systems, boosting the efficiency of wind energy conversion processes, and augmenting the efficacy of solar power applications [1]. Projections suggest that, under a net-zero emission scenario by 2050, the demand for critical materials, encompassing REEs, is anticipated to escalate by a factor of 16 compared to 2020 levels [2]. By that juncture, electric motors in electric vehicles are projected to surpass wind turbines as the primary consumers of REEs. Given the non-renewable nature and strategic importance of REEs, securing a sustainable and reliable supply chain has emerged as a paramount challenge.

REEs exist in nature mainly in three forms: independent minerals, ion adsorption, and isomorphic substitution [3]. Currently, the industrial sector mainly depends on the first two forms mentioned above to fulfill its demand for rare earth elements (REEs) [4]. However, due to the continuous growth in demand and the ongoing exploitation of rare earth mines, the deposits of REEs in the form of independent minerals are being gradually depleted. As a consequence, the development and utilization of co-occurring and associated REE resources have become a research focus. Although REE resources in the form of isomorphic substitution are abundant, their low and dispersed content in associated minerals makes the recovery of REEs from industrial wastes of associated mines not only more reasonable but also economically beneficial [5].

Phosphogypsum (PG) is an industrial waste generated during the production of phosphoric acid via the sulfuric acid process, with 4.5 to 5.5 tons of phosphogypsum produced for every ton of phosphoric acid [6]. As of 2024, the annual global production of PG has exceeded 100 million tons, and due to low recycling rates, over 6 billion tons have accumulated. The REE content in PG ranges from 0.01 to 0.60 wt% [7], meaning that these accumulated PG deposits contain over 1 million tons of REEs, a quantity that even exceeds the total global REE production in the past three years. Therefore, developing efficient and economical extraction technologies for REEs from these industrial wastes has become a research hotspot.

Since the late 20th century, most countries have begun researching the extraction of REEs from solid wastes such as PG, with the primary extraction method being chemical leaching using inorganic strong acids (H_2_SO_4_, HNO_3_, etc.) [8,9]. This method involves adjusting parameters like acid concentration (1–5 mol/L), temperature (30–80 °C), leaching time (1–48 h), and liquid-to-solid ratio, which significantly affect extraction efficiency. Reported extraction rates vary from 50% to 90%, depending on the conditions and specific REEs. After extensive research and practical applications, this method has reached a relatively mature stage. However, using strong acids to leach REEs from PG increases the acidity of the PG, significantly affecting its subsequent comprehensive utilization and posing severe environmental impacts [10].

Bioleaching is a technology that utilizes microorganisms and their metabolites to extract metal elements from minerals or industrial wastes. Compared with traditional pyrometallurgy and hydrometallurgy, bioleaching offers advantages such as environmental friendliness and lower costs [11]. In the field of REEs extraction, bioleaching technology has attracted increasing attention due to its potential sustainability [12].

Numerous studies have demonstrated that specific bacteria and fungi are capable of efficiently leaching REEs [13]. For instance, *Desulfovibrio desulfuricans* can leach REEs from PG, with a leaching efficiency of up to 80% for Y [14]. Additionally, Yang Qu et al. isolated a fungus, *Penicillium tricolor*, from red mud and used this strain to leach REEs and radioactive elements (Th and U) from red mud. The extraction efficiency for Th reached 45.0% in the two-step process at 5% and 10% pulp densities [15]. Besides leaching ability, studies have also revealed the adsorption capacity of microorganisms for rare earth metal ions. For instance, *Bacillus subtilis* adsorbs 15 lanthanide REEs ions through its cell wall, with an adsorption capacity of 1100 μmol/g for trivalent lanthanide rare earth metal ions at pH values between 2.5 and 4.5 [16].

Previously, our research group conducted a study on the leaching of PG by employing the fungus *Aspergillus niger* and achieved a comprehensive leaching efficiency of 74% [17]. Considering that bacteria, due to their shorter generation times and higher mutation rates, tend to adapt rapidly to changing environmental conditions, we were interested in exploring the bioleaching efficiency of a bacterial culture for phosphogypsum-leaching [18]. *G. oxydans*, a strictly aerobic Gram-negative bacterium belonging to the Acetobacteraceae family [19], secretes a bioleaching solution rich in gluconic acid in the presence of glucose [20]. Therefore, this study selected *G. oxydans* as the target strain to investigate its leaching effect on REEs in PG and study the influence of different leaching conditions on REEs leaching efficiency. By analyzing the changes during the leaching process, we aim to reveal the leaching mechanism of REEs from PG by *G. oxydans* and the enrichment pattern of REEs in the leaching solution, providing a theoretical basis and practical application example for the efficient and environmentally friendly comprehensive utilization of PG, the comprehensive recovery of REEs from PG, and achieving a balance between the production and consumption of PG.

## 2. Results and Discussion

### 2.1. Study on the Phase Composition and Components of Phosphogypsum

**Figure 1:**
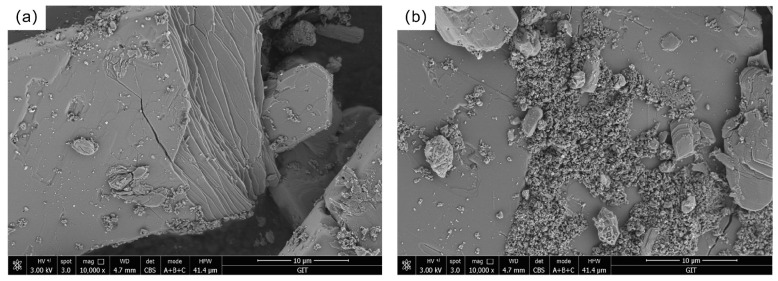
SEM images of PG: (**a**) Fracture Surface; (**b**) Material Surface.

The PG sample used in this experiment was sourced from the Xifeng Phosphorus Mine in Guizhou Province, China. The PG was ground into powder using an agate mortar, passed through a 200-mesh standard sieve, and dried for sample preparation. Figure 1 visually presents the scanning electron microscope (SEM) images of the PG. Through in-depth analysis of the SEM images, we found that the PG sample exhibits a partially faulted structure, characterized by plate-like crystalline forms, which are primarily composed of CaSO_4_·2H_2_O. Additionally, numerous small particles are aggregated on the surface, and these particles may include calcium fluoride, among other substances.

**Figure 2:**
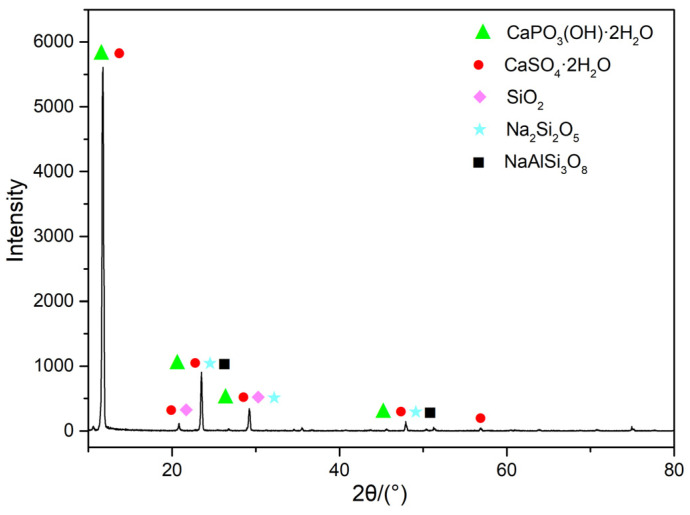
XRD pattern of PG. The peaks correspond to the following phases: CaPO_3_(OH)·2H_2_O (PDF No. 41-1480), CaSO_4_·2H_2_O (PDF No. 33-0311), SiO_2_ (PDF No. 46-1027), Na_2_Si_2_O_5_ (PDF No. 33-0108), and NaAlSi_3_O_8_ (PDF No. 9-0466).

The elemental results determined by X-ray fluorescence (XRF) are shown in Table 1. The measured contents of SO_3_ and CaO were determined to be 50.73% and 37.73%. Considering the elemental composition and content in Table 1, it is confirmed that the primary mineral component of PG is CaSO_4_·2H_2_O. The analysis also identifies that the main trace components are SiO_2_, F, Al_2_O_3_, and Fe_2_O_3_. Further phase analysis was conducted on the original mineral sample. Combining the data in Table 1 with the information from Figure 2, it can be seen that the sample contains minerals such as CaSO_4_·2H_2_O, CaPO_3_(OH)·2H_2_O, SiO_2_, and NaAlSi_3_O_8_.

The REEs content analyzed by inductively coupled plasma mass spectrometry (ICP-MS) is presented in Table 2, showing that the total REEs content in the experimental sample is 77,280 μg/kg, with Yttrium (Y), Neodymium (Nd), Cerium (Ce), and Lanthanum (La) being the dominant elements, accounting for 72.43% of the total.

### 2.2. Growth Characteristics of Gluconobacter oxydans

#### 2.2.1. Growth Curve of *G. oxydans*

**Figure 3:**
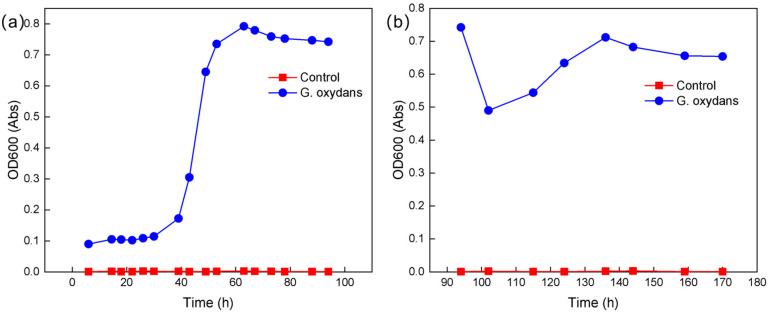
Growth curve of *G. oxydans*: (**a**) before adding nutrients; (**b**) after adding nutrients.

The concentration of microorganisms in a suspension is linearly related to its absorbance at 600 nm. Therefore, the growth curve of *G. oxydans* can be plotted based on the absorbance of the bacterial suspension [21]. The *G. oxydans* is cultured in *Acetobacter* culture medium (Section 3.2). The *G. oxydans* fermentation broth was diluted 10-fold, and the absorbance at 600 nm was measured and recorded as OD600 to plot the growth curve. As shown in Figure 3, *G. oxydans* exhibits a lag phase from 0 to 30 h, followed by a logarithmic growth phase from 30 to 64 h with vigorous metabolism, reaching a maximum absorbance of 0.792. After 64 h of cultivation, *G. oxydans* enters a stationary growth phase, with gradual accumulation of metabolites and slower growth.

When bacterial growth reaches its peak, nutrients (0.1 g/L Glucose solution) equivalent to 1/2 the volume of the original fermentation broth are added, aiming to enhance the continuous fermentation capacity of *G. oxydans* and increase its metabolic product yield through secondary nutrient supplementation. Following the original procedure, the secondary growth curve of the strain is then plotted [22]. As shown in Figure 3b, the absorbance drops to 0.44 after adding nutrients at 99 h, due to the increase in solution volume to 1.5 times. Between 99 h and 136 h, the growth curve shows continuous growth, and after 136 h, the growth rate slowly declines, entering the stationary phase.

#### 2.2.2. Relationship Between pH of *G. oxydans* Culture and Cultivation Time

In studies on microbial leaching of metals or REEs from minerals, pH has a significant impact on leaching efficiency [22]. Microorganisms consume nutrients in the medium during growth and produce organic acids, metabolites, and extracellular polymers, which can alter the pH of the leaching system and in turn affect microbial growth [23]. Therefore, monitoring pH changes is crucial for understanding microbial growth characteristics and metabolic pathways.

Organic acids produced during microorganisms’ metabolism (such as oxalic acid, citric acid, and ketoglutaric acid) play an important role in the migration process of metal ions [24]. These small organic molecules can combine with metal elements to form coordination complexes, reducing the saturation of metal elements in the solution and thus promoting mineral dissolution [25,26]. In harsh growth environments, microorganisms release organic acids to dissolve nearby minerals to obtain nutrients [27]. Furthermore, the normal growth and metabolic processes of microorganisms can vary with changes in nutrient supply, which may affect their efficiency and selectivity in the bioleaching process [28].

**Figure 4:**
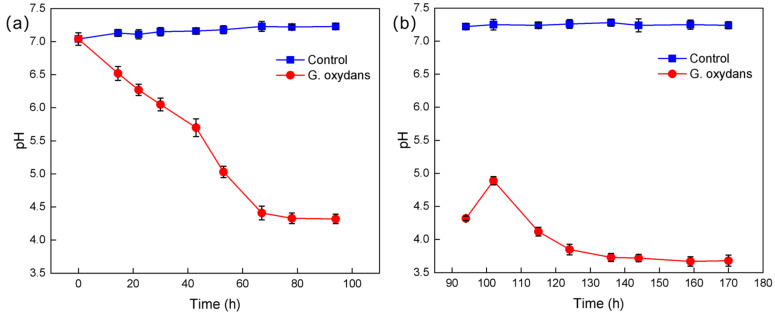
*G. oxydans*’ pH change graph: (**a**) before adding nutrients; (**b**) after adding nutrients.

During the growth of *G. oxydans*, changes in pH are closely related to the production of organic acids. As shown in Figure 4a, the pH of *G. oxydans* cultures continuously decreases during growth, reaching a minimum of 4.3 at 73 h and then stabilizing. This is due to the continuous production of organic acids (such as oxalic acid, lactic acid, citric acid, etc.) by *G. oxydans*, leading to a rapid decrease in pH. From Figure 4b, it can be seen that after the stable growth of *G. oxydans*, nutrients (0.1 g/L glucose solution) equivalent to 1/2 the volume of the original fermentation broth are added. Then, at 159 h, the pH reaches a second minimum of 3.67. Comparatively, the minimum pH of 3.67 in the second growth cycle is significantly lower than that in the first phase, indicating that after adding nutrients in the second growth cycle, the microorganisms begin a new round of growth and metabolism. The accumulation rate of organic acids increases after stabilizing at the end of the first growth cycle, leading to the pH reaching its lowest point again.

### 2.3. Impact of Leaching Conditions on REEs

During the microbial leaching process, numerous factors are known to exert an influence on leaching efficiency, among which temperature (20–44 °C), agitation speed (80–150 rpm), liquid-to-solid ratio (2:1–16:1), and inoculum size are of particular significance. In this experiment, the focus has been placed on the three key factors, namely temperature, agitation speed, and liquid-to-solid ratio, with the aim of investigating their respective impacts on the leaching efficiency of REEs.

#### 2.3.1. Leaching Temperature

**Figure 5:**
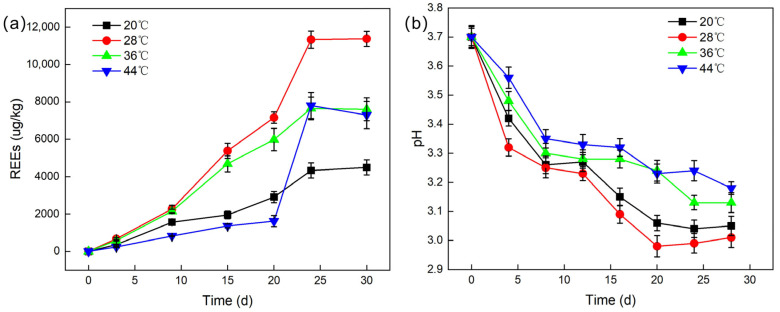
The influence of different temperatures on the bioleaching efficiency (**a**) and pH (**b**) of REEs.

Leaching temperature is a crucial factor affecting the leaching efficiency of REEs from PG. Experiments were conducted with an inoculum size of 10%, a liquid-to-solid ratio of 2:1, and an agitation speed of 150 rpm, at temperatures of 20 °C, 28 °C, 36 °C, and 44 °C. The pH values during the leaching experiments were recorded. The effect of temperature on REE leaching is shown in Figure 5.

Figure 5a shows that temperature significantly affects the leaching of REEs by *G. oxydans*. Starting from 20 °C, the leaching efficiency increases with temperature, peaking at 28 °C, and then decreases. At 28 °C, the leaching efficiency reaches its optimum at 16.5%, with a maximum total leached REEs content of 12,048.49 μg/kg after about 22 days. This coincides with the optimal temperature for *G. oxydans* [29], where its reproduction and metabolism are more rapid, producing more organic acids, metabolites, and extracellular polymers.

Figure 5b indicates that higher microbial activity leads to a lower pH. At 28 °C, the pH drops to 2.98, even lower than the minimum pH in Section 2.1, possibly due to the acidic nature of the PG leachate. Comparing Figure 5a,b, we can see that changes in pH do not exactly correlate with the total leached REE content. High or low temperatures can affect microbial growth [30]. At low temperatures, reduced microbial activity leads to slower leaching, with lower organic acid production. At high temperatures, while the pH decreases, microbial activity also declines, and the dissolved oxygen may lead to the production of acids, such as acetic acid, that are less effective for dissolving refractory REEs [31], thereby lowering leaching efficiency. However, an unexpected increase in REE leaching efficiency was observed between days 20 and 25 at 44 °C. This could be due to a microbial adaptation process, where *G. oxydans* gradually adjusted to the higher temperature, leading to a recovery in metabolic activity and enhanced production of more effective acids, which, in turn, improved the leaching efficiency [32].

#### 2.3.2. Agitation Speed

The leaching efficiency under different agitation speeds was studied. With an inoculum size of 10%, a temperature of 28 °C, and a liquid-to-solid ratio of 2:1, agitation speeds of 80 rpm, 100 rpm, 130 rpm, and 150 rpm were set, and the pH values during leaching experiments were recorded.

**Figure 6:**
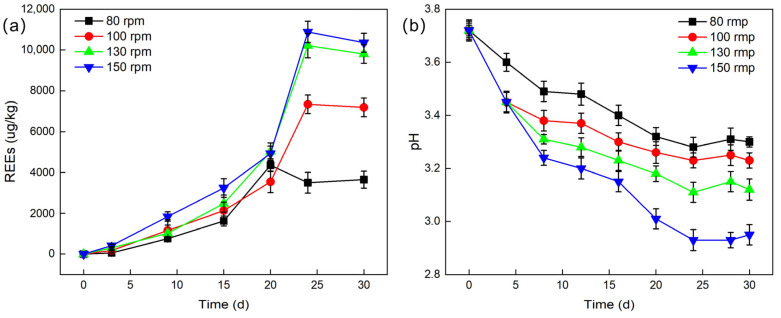
The influence of different agitation speed on the leaching effect (**a**) and pH (**b**) of REEs.

As shown in Figure 6a, as the agitation speed increases from 80 rpm to 130 rpm, the leaching efficiency of REEs significantly increases. Although the leaching efficiency still improves somewhat when the agitation speed is further increased to 150 rpm, the increase is relatively small. At an agitation speed of 150 rpm, the cumulative leached REE content peaks at 10,884.74 μg/L, corresponding to a leaching efficiency of 14.9%. This value represents the optimal leaching outcome under the investigated agitation speed conditions. This result may be attributed to the following aspects. Firstly, the activity of *G. oxydans* increases with the agitation speed. During the rotation and agitation of the culture medium, the fermentation broth continuously washes the walls of the Erlenmeyer flask, creating a larger contact surface between the culture medium and air at a reasonable and higher agitation speed, allowing more oxygen to dissolve in the fermentation broth, thereby promoting the growth and metabolic activities of *G. oxydans* [33]. Secondly, a higher agitation speed ensures sufficient contact between *G. oxydans* and PG particles, increasing the opportunities for it to adsorb REEs. At the same time, the organic acids, metabolites, and extracellular polymers produced by *G. oxydans* effectively disrupt the crystal structure of PG through acidolysis and complexation reactions, further improving the leaching efficiency of REEs. In addition, as shown in Figure 6b, the pH value also shows a decreasing trend with the increase in agitation speed, which is consistent with the previously observed pattern, further verifying the impact of agitation speed on the leaching process.

#### 2.3.3. Ratio of Leaching Solution Volume to PG Mass

Studies were conducted on the impact of various liquid-to-solid ratios on the total amount of leached REEs. With controlled inoculum size, temperature, and agitation speed, liquid-to-solid (L/S) ratios of 2:1, 4:1, 8:1, and 16:1 were set, and the pH values during leaching experiments were recorded.

**Figure 7:**
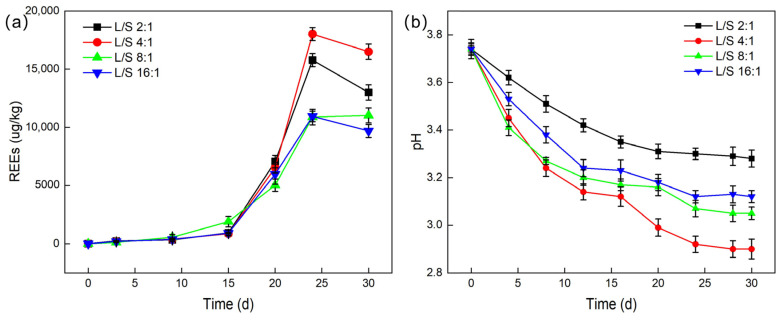
The influence of different liquid–solid ratios on the leaching effect (**a**) and pH (**b**) of REEs.

**Figure 8:**
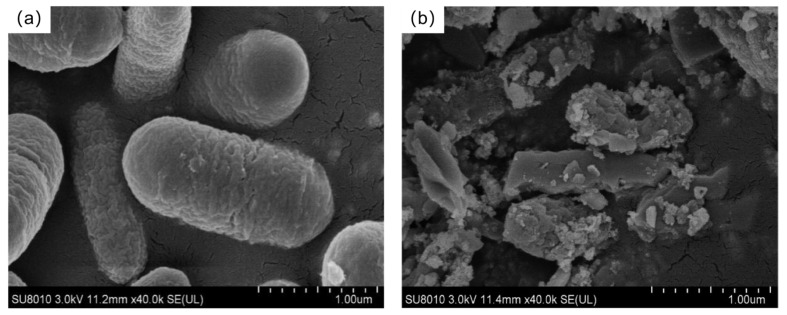
SEM images of *G. oxydans*: (**a**) original; (**b**) after leaching (L/S = 2:1).

As shown in Figure 7, the leaching efficiency of REEs is most significant at a liquid-to-solid ratio of 4:1, with the total leached REE content reaching a maximum of 18,020.3 μg/L and a leaching efficiency of up to 24.67%. The impact of different liquid-to-solid ratios on the total amount of leached REEs shows significant differences, with the order being: 4:1 > 2:1 > 16:1 ≈ 8:1. This result indicates that an appropriate liquid-to-solid ratio can promote sufficient contact between *G. oxydans* and PG particles, facilitating the adsorption or dissolution of rare earth ions. However, when the liquid-to-solid ratio is too high (such as 2:1), it may cause severe damage to the structure of *G. oxydans*, which negatively affects its growth and metabolism [34], thereby reducing the leaching efficiency of REEs. This was further confirmed by scanning electron microscopy (SEM) observations, which revealed that the structure of *G. oxydans* was severely damaged by PG when the liquid-to-solid ratio was 2:1 (Figure 8).

### 2.4. Relationship Between Leaching Efficiency of REEs, pH, and Leachants

As has been previously analyzed, the organic acids and metabolites produced by microorganisms during their growth have a significant impact on the pH of the system, thereby altering the leaching efficiency of REEs. To delve deeper into this complex factor, we directly regulated the pH of the leaching system and compared the effects of different leachants on the leaching efficiency of various REEs.

**Figure 9:**
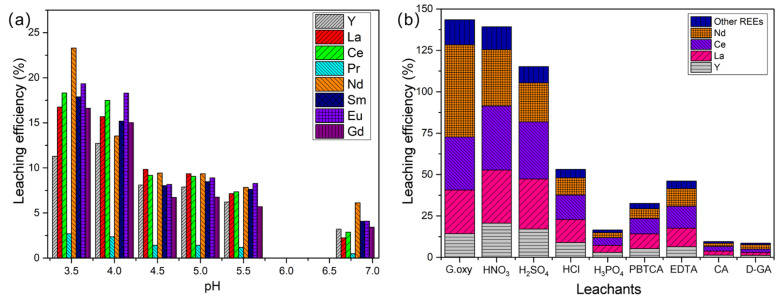
(**a**) The influence of different pH values on the leaching efficiency of each REE. (**b**) The effect of the type of leaching agent on the leaching efficiency of each REE.

Under conditions of a liquid-to-solid ratio of 2:1, an agitation speed of 150 rpm, and a temperature of 28 °C, the pH of the medium was set to 3.5, 4, 4.5, 5, and 5.5. A control group with a pH of 6.8 was established without the addition of *G. oxydans*, and the leaching duration was 30 days for all groups. As shown in Figure 9a, lower pH values generally improved the leaching efficiency of REEs in PG. Although most REEs exhibited better leaching efficiency at pH 3.5, the leaching efficiency of each element did not change uniformly with pH. For example, the leaching efficiency of Pr remained at a low level across all pH values (up to only 2.7%), while Y reached its highest leaching efficiency at pH 4. Notably, the leaching efficiency of Nd significantly increased to 23.29% at pH 3.5. These results indicate significant differences in the leaching mechanisms of different REEs.

Upon further comparison of the effects of different leachants on the leaching amount of various REEs (Figure 9b), we found that *G. oxydans*, with controlled reaction conditions, exhibited comparable leaching efficiency to inorganic strong acids and had a clear advantage over simple organic weak acids. Specifically, the highest leaching efficiencies for Y, La, and Ce were observed using nitric acid, with values of 20.66%, 32.10%, and 38.63%, respectively. *G. oxydans* performed well in leaching Nd, with a leaching efficiency of 55.67%. Remarkably, *G. oxydans* showed significantly higher leaching efficiency for Nd than inorganic strong acids like nitric acid and sulfuric acid. Notably, *G. oxydans* demonstrated particularly high efficiency in leaching Nd, achieving a leaching efficiency of 55.67%, which was comparable to that achieved with 3 M HNO_3_ (57.5%) and 3 M HCl (52.0%) under heating conditions (50 °C), while H_2_SO_4_ (3 M) achieved only 21.3% for Nd, as reported by Walawalkar et al. [9].

This finding suggests that the leaching process of REEs by *G. oxydans* involves not only ion migration and organic acid complexation but also potentially more complex leaching mechanisms [35]. Importantly, the pH of the solution is a critical factor influencing the efficiency of bioleaching. Therefore, bioleaching technology demonstrates potential advantages in selective leaching and specific adsorption for REEs with different characteristics [36].

### 2.5. Analysis of the System After Leaching

After the bioleaching process by *G. oxydans*, the system yielded both a leachate containing REEs and the residual phosphogypsum (PG). These analyses revealed significant structural transformations in PG, providing detailed insights into the mechanisms by which *G. oxydans* interacts with PG during leaching. To further investigate the changes in element content and morphology of PG after leaching with *G. oxydans*, we conducted X-ray diffraction (XRD), X-ray fluorescence (XRF), and scanning electron microscopy (SEM) analyses on samples from the leaching system.

**Figure 10:**
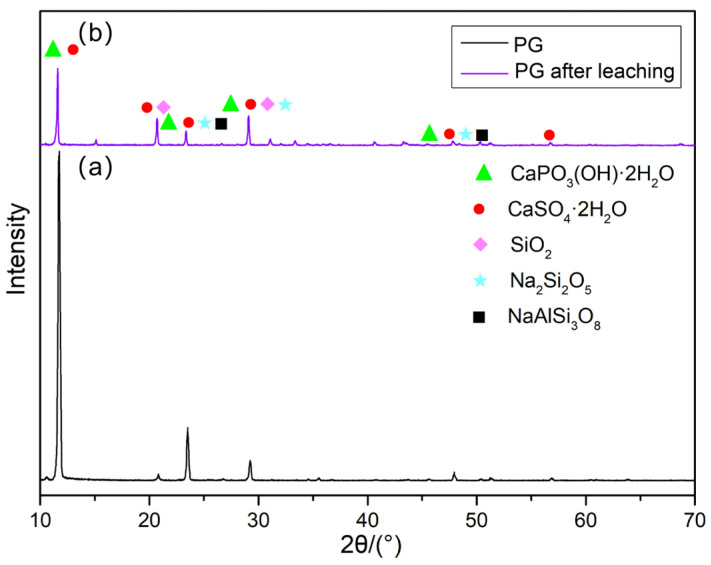
XRD pattern: (**a**) PG, (**b**) PG after leaching. The peaks correspond to the following phases: CaPO_3_(OH)·2H_2_O (PDF No. 41-1480), CaSO_4_·2H_2_O (PDF No. 33-0311), SiO_2_ (PDF No. 46-1027), Na_2_Si_2_O_5_ (PDF No. 33-0108), and NaAlSi_3_O_8_ (PDF No. 9-0466).

Referring to Figure 10, there is a noticeable difference in the XRD patterns of PG before and after leaching. After microbial leaching, the diffraction peak intensity at 12° for dicalcium phosphate dihydrate and gypsum significantly decreased, and the diffraction peak intensity at 23° for albite and Na_2_Si_2_O_5_ also decreased markedly. This change indicates that the metabolic substances produced by *G. oxydans* during its growth effectively disrupted the crystal structure of PG through complexation and acidolysis, transforming it into an amorphous state. This structural change is highly beneficial for the leaching of REEs, enhancing leaching efficiency [37].

Further comparison of the major element contents in PG before and after leaching (as shown in Table 3) revealed that some non-hazardous elements, such as Si, Al, and Fe, were leached to some extent. For instance, the content of SiO_2_ decreased from 4.97% in the original sample to 1.444% after leaching. Similarly, the contents of Al_2_O_3_ and Fe_2_O_3_ decreased from 0.75% and 0.59% to 0.287% and 0.189%. On the other hand, harmful elements like P_2_O_5_ and F were also leached during the process, with P_2_O_5_ decreasing from 0.91% in the original sample to 0.788%, and the content of F even dropped below the detection limit. These results indicate that while some non-hazardous elements are leached alongside the REEs, potentially harmful elements such as F and P_2_O_5_ are also removed, providing favorable conditions for the subsequent environmentally friendly applications of PG.

**Figure 11:**
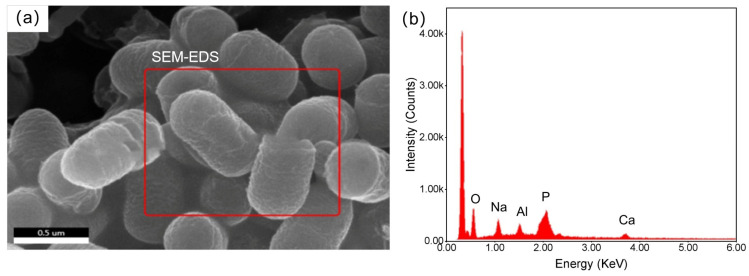
SEM (**a**) and EDS (**b**) of original *G. oxydans*.

**Figure 12:**
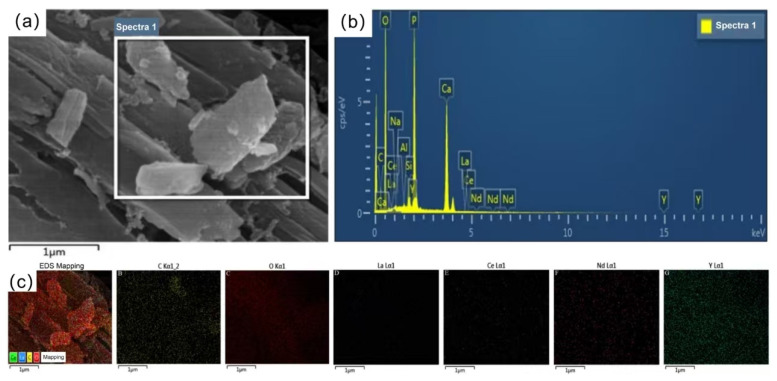
SEM (**a**), EDS (**b**), and EDS-Mapping (**c**) of *G. oxydans* after leaching.

Figure 11 shows the morphology and elemental composition of *G. oxydans* before the leaching experiment with PG. The *G. oxydans* without exposure to PG had a smooth, uniformly wrinkled, and opaque surface with neat edges and sharp corners. The colonies were circular and rod-shaped, and the bacterial structure was intact. The strain mainly contained C, O, Na, Al, P, and Ca elements. However, significant changes occurred on the microscopic surface of *G. oxydans* after the leaching experiment (as shown in Figure 12). The surface of *G. oxydans* exhibited “skin” loss or small pits of varying sizes, the bacterial size decreased, and various small fragments from the decomposition of PG were adsorbed on the surface. Some of these fragments formed granular clusters with blurred edges. Combined with SEM-EDS analysis (Figure 11b and Figure 12b), we found that the surface of *G. oxydans* adsorbed various REEs, including Y, Nb, Ce, and La, after leaching PG, albeit with varying relative contents but a rich variety of adsorbed elements. These analyses revealed significant structural transformations in PG, providing detailed insights into the mechanisms by which G. oxydans interacts with PG during leaching.

The post-treatment of the leachate after rare earth element (REE) extraction is also an important aspect to consider. Currently, we are experimenting with the addition of precipitants (e.g., oxalic acid) to the REE-containing leachate to precipitate the REEs. Following this, the nutrient medium is replenished, and *G. oxydans* is cultured again to re-leach the REEs from the phosphogypsum. This approach aims to reduce waste-liquid generation. Furthermore, future research will focus on separating mixed REE precipitates to achieve better resource recovery.

## 3. Experimental Materials and Methods

### 3.1. Experimental Reagents

Deionized water was used throughout the experiments. The reagents included BR yeast extract powder (Beijing, China), AR glucose (C_6_H_12_O_6_·H_2_O, (Tianjin, China)), AR calcium carbonate (CaCO_3_, (Shantou, China)), biological reagent agar powder (Beijing, China), AR anhydrous ethanol (CH_3_CH_2_OH, (Chongqing, China)), AR/GR nitric acid (HNO_3_, (Chongqing/Shanghai, China)), and AR pure sodium chloride (NaCl, (Shantou, China)), among others.

*Gluconobacter oxydans* (*G. oxydans*) was purchased from Beijing Bio-Tech Pack Technology Co., Ltd., (Beijing, China).

### 3.2. Leaching Experiment

The medium used for the leaching experiment was an *Acetobacter* Culture medium, with its components and concentrations listed in Table 4. The prepared liquid medium was adjusted to pH 6.8 using 20% diluted sulfuric acid and 10% NaOH.

The leaching experiment of PG by *G. oxydans* was conducted in an oscillator to investigate the effects of liquid-to-solid ratio, agitation speed, and temperature on REE leaching. The leaching experiments were carried out in 250 mL Erlenmeyer flasks, which were autoclaved at 121 °C for 20 min. The experimental system consisted of leaching solution, to which a preset concentration of leaching agent and PG samples were added according to a specific liquid-to-solid ratio. Glucose solution (0.1 g/L) was added in two stages, with the first 100 mL added at the beginning of the experiment and the second 50 mL added later to supplement nutrition during the experiment. To prevent evaporation of the liquid during the leaching experiment, the mouths of the Erlenmeyer flasks were plugged with breathable rubber stoppers that allow for gas exchange while minimizing liquid loss. The flasks were then placed in a vertical single-door full-temperature oscillator for constant-temperature agitation culturing at 28 °C and a speed of 150 rpm. Approximately 2 mL of leaching solution was collected at predetermined time intervals using a pipette, followed by centrifugation to obtain the supernatant. REE content was tested using ICP-MS, and the pH value of the remaining leaching solution after centrifugation was measured. After leaching, a centrifuge was used to separate the solid and liquid phases of the sample. Part of the leached residue was dried and ground into powder for later use. Scanning electron microscopy (SEM), EDS energy spectrum, X-ray diffraction (XRD), and X-ray fluorescence (XRF) were used to characterize the samples, observe the surface morphology of PG before and after leaching, analyze the elemental composition, and examine the distribution of bacteria on the surface of PG.

### 3.3. Analytical and Testing Methods

A PHS-3C model pH meter (Shanghai INESA Scientific Instrument Co., Ltd., (Shanghai, China)) was used to analyze the pH value of the experimental system before and during leaching, exploring the pH changes during the leaching of REEs from PG by the strain. A UV2400 model UV-visible spectrophotometer (Ningbo Sunny Optical Hengping Scientific Instrument Co., Ltd., (Ningbo, China)) was used to measure the growth curve of the strain, grasping the growth and change process of the strain. The NexION 2000 model inductively coupled plasma mass spectrometer (ICP-MS) from PerkinElmer, Shelton, CT, USA, was used to measure the REE content in the leaching solution. A suite of advanced analytical tools, including X-ray diffraction (XRD, Ultima IV, Tokyo, Japan), X-ray fluorescence spectrometry (XRF, CNX-808, Beijing, China), thermal field emission scanning electron microscopy with energy dispersive spectroscopy (SEM-EDS, Nova Nano SEM 450, Tucson, AZ, USA), and cold field emission scanning electron microscopy (SEM, Hitachi SU8010, Tokyo, Japan), was used to analyze and measure the changes in mineral structure and elemental composition, surface microstructure, and bacterial micromorphology before and after the leaching experiment of PG, exploring the reaction pattern of REEs in PG and the enrichment pattern of REEs in the leaching solution.

## 4. Conclusions

Through a series of systematic experiments, this study focused on the bioleaching of REEs from phosphogypsum using Gluconobacter oxydans (*G. oxydans*) through systematic experiments, leading to the following core conclusions.

The growth curve of *G. oxydans* conforms to bacterial growth patterns, with the organic acids and metabolic substances produced by its metabolism causing a continuous decrease in the pH value of the leaching system. Experimental results indicate that *G. oxydans* has efficient leaching capability for REEs in phosphogypsum, particularly under a liquid-to-solid ratio of 4:1, where the maximum total leached REEs content reached 18,020.3 μg/L after approximately 21 days, with a maximum leaching efficiency of 24.67%. Notably, *G. oxydans* achieved a leaching efficiency of 55.67% for Nd, which is comparable to values reported for traditional acid leaching methods, such as 57.5% using 3 M nitric acid under heating conditions. Further analysis revealed that the decrease in pH significantly promoted the leaching of REEs, especially for Nd, which showed a significant increase in leaching efficiency when the pH dropped to 3.5. This suggests that the leaching of elements is not only related to complexation but is also strongly pH dependent. Through characterization methods such as XRD, SEM, and EDS, we confirmed that the surface structure of *G. oxydans* was disrupted during the leaching process and successfully adsorbed various REEs including Y, Ce, La, and Nd.

In conclusion, this study not only verified the feasibility and efficiency of bioleaching REEs from phosphogypsum using *G. oxydans* but also provided new ideas and methods for the comprehensive utilization of phosphogypsum and the sustainable supply of REEs. Future research can further optimize leaching conditions and deeply explore the synergistic mechanisms of *G. oxydans* with other microorganisms to promote the industrial application and development of this technology.

## Figures and Tables

**Table 1:** Contents of major elements in Xifeng PG.

Content	SO_3_	CaO	SiO_2_	F	P_2_O_5_	Al_2_O_3_	Fe_2_O_3_	SrO	BaO	Other
wt%	50.73	37.73	4.97	3.96	0.91	0.75	0.59	0.071	0.18	0.109

**Table 2:** The content of REEs in Xifeng PG.

Element	Sc	Y	La	Ce	Pr	Nd	Sm	Eu
μg/kg	-	24,560	8380	13,160	10,480	9880	2340	600
Element	Gd	Tb	Dy	Ho	Er	Tm	Yb	Lu
μg/kg	3640	-	2440	-	-	620	1180	-

**Table 3:** Main elements’ content of PG after leaching experiment (L/S = 2:1, 150 rpm, 28 °C).

Content (wt%)												
PG Sample	SO_3_	CaO	SiO_2_	F	Na_2_O	Cl	P_2_O_5_	Al_2_O_3_	Fe_2_O_3_	SrO	BaO	Other
Original	50.73	37.73	4.97	3.96	-	-	0.91	0.75	0.59	0.071	0.18	0.109
After leaching	52.55	42.31	1.444	-	1.293	0.962	0.788	0.287	0.189	0.078	-	0.099

**Table 4:** Composition of *Acetobacter* culture medium.

Content	Glucose	CaCO_3_	Yeast Extract
g/L	0.1	0.02	0.01

## Data Availability

Data is contained within the article.
